# Beyond antiplatelets: The role of glycoprotein VI in ischemic stroke

**DOI:** 10.1177/1747493016654532

**Published:** 2016-06-16

**Authors:** Isuru Induruwa, Stephanie M Jung, Elizabeth A Warburton

**Affiliations:** 1 Department of Clinical Neurosciences, Box 83, Cambridge University Biomedical Campus, Cambridge, UK; 2 Department of Biochemistry, University of Cambridge, Cambridge, UK

**Keywords:** Stroke, platelets, glycoprotein VI, antiplatelets

## Abstract

**Background:**

Platelets are essential to physiological hemostasis or pathological thrombus formation. Current antiplatelet agents inhibit platelet aggregation but leave patients at risk of systemic side-effects such as hemorrhage. Newer therapeutic strategies could involve targeting this cascade earlier during platelet adhesion or activation via inhibitory effects on specific glycoproteins, the thrombogenic collagen receptors found on the platelet surface.

**Aims:**

Glycoprotein VI (GPVI) is increasingly being recognized as the main platelet-collagen receptor involved in arterial thrombosis. This review summarizes the crucial role GPVI plays in ischemic stroke as well as the current strategies used to attempt to inhibit its activity.

**Summary of review:**

In this review, we discuss the normal hemostatic process, and the role GPVI plays at sites of atherosclerotic plaque rupture. We discuss how the unique structure of GPVI allows for its interaction with collagen and creates downstream signaling that leads to thrombus formation. We summarize the current strategies used to inhibit GPVI activity and how this could translate to a clinically viable entity that may compete with current antiplatelet therapy.

**Conclusion:**

From animal models, it is clear that GPVI inhibition leads to an abolished platelet response to collagen and reduced platelet aggregation, culminating in smaller arterial thrombi. There is now an increasing body of evidence that these findings can be translated into the development of a bleeding free pharmacological entity specific to sites of plaque rupture in humans.

## Introduction

Ischemic stroke is a worldwide leading cause of disability and death.^1^ An important and distinct subtype of ischemic stroke is due to the acute rupture of atherosclerotic plaques seen in large artery disease.^2^ The mechanism of cerebral hypoperfusion involves platelets binding to exposed sub-endothelial collagen (adhesion), resulting in platelet activation, aggregation, and thrombus formation. Unstable thrombi can detach and travel to cerebral vessels causing stroke. As a result, the current management of acute stroke involves using antiplatelet agents such as aspirin and clopidogrel that inhibit platelet activation/aggregation, but often at the risk of off-target adverse effects such as hemorrhage.^3,4^

Novel therapeutic strategies could involve targeting this cascade earlier during platelet adhesion or activation via inhibitory effects on specific platelet glycoproteins, the thrombogenic collagen receptors on their surfaces. Glycoprotein VI (GPVI) is one such crucial transmembrane collagen receptor and pharmacological inhibition of GPVI, in order to stop pathological thrombus formation specific to the site of vessel injury, is currently being explored.

## Hemostasis – clotting and thrombus formation

Platelets are anucleate cells derived from megakaryocytes. They contain unique cytoplasmic structures, α- and dense granules that can rapidly release their contents upon activation, promoting thrombus formation. Under normal hemostasis numerous protective barriers to thrombus formation exist to contain it to the injured site. This includes the continuous lining of endothelium that prevents platelets coming into contact with the prothrombotic sub-endothelial matrix,^5^ expression of ectonucleoside triphosphate diphosphohydrolase (CD39/ENTPD1)^6^ and secretion of prostacyclin (PGI2) and nitric oxide.

There are four main platelet glycoprotein receptors that participate in the platelet–collagen interaction and facilitate thrombus formation. The GPIb-IX-V complex (GPlbα, GPIbβ, GPIX and GPV) binds to von Willebrand factor (VWF) immobilized on collagen. Both GP Ia/IIa (integrin α_2_β_1_) and GPVI bind directly to exposed collagen. GPIIb/IIIa (integrin α_IIb_β_3_) are converted to their high-affinity forms via inside-out signaling in activated platelets, enabling them to bind free fibrinogen and VWF.

**Figure 1.**
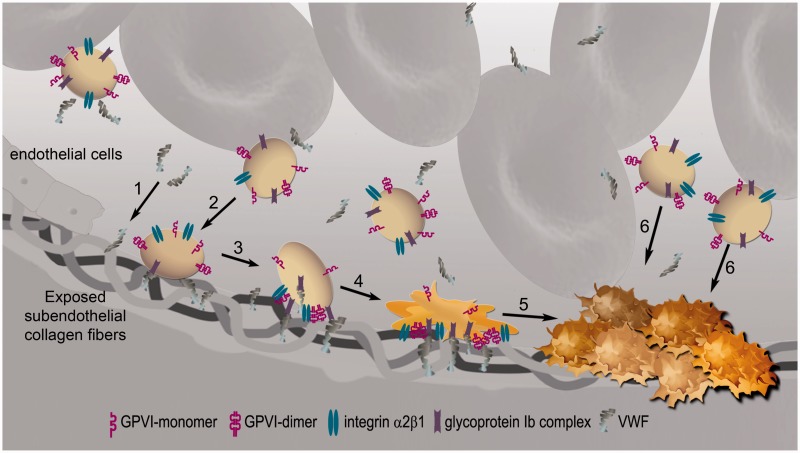
Collagen-binding receptors (GPIb, GPVI, and integrin α2β1) involved in platelet adhesion and activation. Sub-endothelial collagen exposed upon vessel injury binds to von Willebrand factor (vWF) in the blood (step 1). Platelets become tethered to collagen fibers by their vWF receptor GPIb—a weak interaction—so platelets transiently bind and detach, moving along the collagen (step 2). Platelets become firmly attached when their collagen receptors GPVI dimer and integrin α2β1 binds to collagen; signaling through either GPVI or GPIb converts integrin α2β1 to its high affinity form (step 3). GPVI engagement with collagen initiates a signaling cascade that culminates in platelet activation, spreading, and granule contents release (step 4), recruiting other platelets and forming a thrombus (steps 5 and 6). Integrin αIIbβ3 (not shown) becomes activated through inside-out-signaling, enabling it to bind fibrinogen, through which inter-platelet bridges can be formed, allowing thrombus propagation.

Fibrous collagen is the ligand for both GPVI and integrin α2β1. Each collagen monomer comprises three 1000 amino acid polypeptides arranged in a triple helix, and bundles of these monomers align to form collagen fibers.^7^ With atherosclerotic plaque rupture, the sub-endothelial fibrillar collagens (types I and III) are exposed to the blood stream. Under high shear (arterial flow), VWF becomes immobilized on the exposed collagen fibers and binds with the GPIb-IX-V complex^8–10^ (Figure 1). Platelets translocate on the collagen surface until firmly arrested through almost simultaneous binding with integrin α2β1 and GPVI. GPVI engagement with collagen (via GPVI dimers) initiates a signaling cascade leading to platelet activation and inside-out signaling (and intracellular calcium mobilization) that converts low affinity forms of integrins α_IIb_β_3_ and α_2_β_1_ into their active forms, as well as further clustering of GPVI receptors.^8^

Activated platelets rapidly synthesize thromboxane A2 (TxA2) and secrete this, along with the contents of their alpha (fibrinogen, P-selectin, and VWF multimers) and dense granules (ADP).^9^ An increase in intracellular calcium in response to ADP and TxA2 induces platelet shape change to an irregular shape with multiple filipoidal surfaces. This facilitates the formation of a close structure of folded platelets in the platelet plug.

Now non-activated platelets are recruited into the growing thrombus, also becoming activated by ADP and TxA2. GPVI-activated platelets provide a pro-coagulant surface for the generation of thrombin—separately through the coagulation cascade by releasing several substances like FV, FXIII, fibrinogen, and Protein S. This further activates platelets and converts fibrinogen to fibrin, resulting in a strong mesh structure.

## Glycoprotein VI and its structure

**Figure 2.**
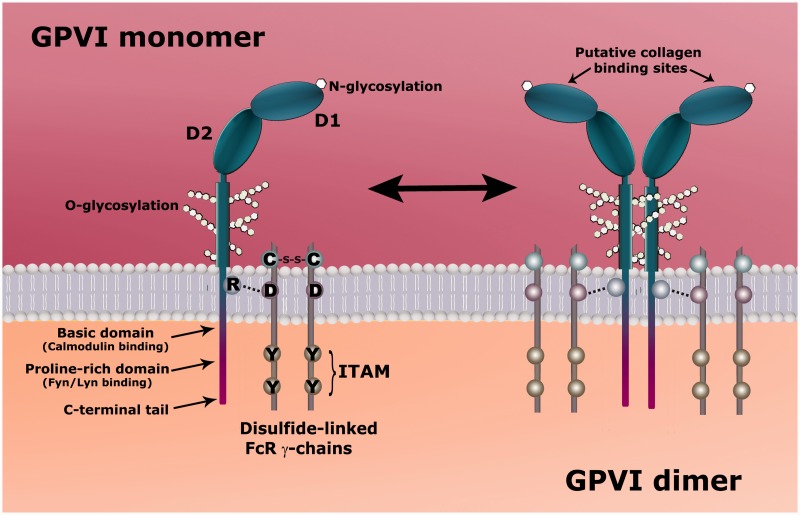
Structures of GPVI monomer and dimer. The extracellular domain of each monomer comprises two IgG domains (D1 and D2) and a mucin-like Ser/Thr-rich domain connecting D1/D2 to the transmembrane domain. The binding site recognizing the GPO triplets of collagen, resides in D1. Each monomer is non-covalently associated through a salt bridge with FcRγ. FcRγ is a disulphide-linked dimer, each chain containing an ITAM sequence, which when phosphorylated binds to the tyrosine kinase Syk. The phosphorylated Syk now initiates signaling. The short intracellular domain of GPVI contains a basic domain that binds to calmodulin; a proline-rich domain that binds to Src kinases Fyn and Lyn, which participate in phosphorylation of ITAM; and a C-terminal tail.

Glycoprotein VI, a 62-kDA transmembrane glycoprotein exclusively expressed in platelets and megakaryocytes, is associated with an immunoreceptor tyrosine-based-activation motif (ITAM) containing signaling subunit FcRγ (Figure 2).^10^ Its gene is mapped to 19q13.4 of the human genome. GPVI contains two IgG-like extracellular domains (D1 and D2) linked by a peptide strand; D2 is connected to the transmembrane domain via a glycosylated stem; and its 51 amino acid cytoplasmic tail is required for signal transmission.^11,12^

GPVI exists in both monomeric and back-to-back dimeric forms on the platelet surface^13^ and forms a non-covalently linked complex with the Fc receptor γ-chain (FcRγ) via a salt bridge formed between their transmembrane domains. FcRγ itself is also a dimer, held together by covalent links and is essential for GPVI expression, as FcRγ knockout mice do not express GPVI.^14^ Dimerization or multimerization of GPVI was first suggested by Berlanga et al.^15^ but proof of the actual existence on platelets was first provided by Jung et al.^16^ Later, they found that constitutive dimers of GPVI make up of about 20% of the total GPVI^13,17^ in resting platelets of normal individuals and platelet activation increases the number of dimers.^17^ Only GPVI dimer has high affinity for collagen, while the monomer binds weakly if at all. Inhibition by dimer specific antibody m-Fab-F markedly inhibited collagen-induced platelet aggregation, highlighting its role as the functional form of GPVI.

## Interaction with collagen and signaling

So how does GPVI interact with the fibrous subendothelial collagen exposed upon vessel injury to induce platelet activation? Much of this data has been gained by using snake venom-derived convulxin and CRP (collagen-related peptide) in vitro, which activate platelets in a similar way to collagen.^7,18^ CRP, a triple helical peptide containing 10 glycine–proline–hydroxyproline (GPO) sequences, is an especially potent platelet agonist specific for GPVI. GPVI was suggested to bind to the GPO sequences in collagen. Smethurst et al.^19^ later found that the minimum recognition motif for GPVI is one GPO.

Using the crystal structure of GPVI and a docking model with CRP, Horii et al.^20^ found that GPVI formed back-to-back dimers, each containing a shallow groove on the D1 domain that is perpendicular to the collagen triple helix, which fits precisely into this 5.5-nm gap.

Loyau et al.^21^ proposed that at least two GPVI dimer-FcRγ complexes are required to create a working signaling unit, as the FcRγ chain is also essential for the function of GPVI and each chain contains an ITAM. Upon GPVI–collagen binding, the Tyr residues of the ITAM sequence become phosphorylated, initiating signal transduction. PCLγ2 is subsequently activated, leading to eventual activation of protein kinase C, resulting in calcium mobilization, degranulation and GPIIb/IIIa activation and the start of the next step in the process—platelet aggregation.

## GPVI shedding and receptor downregulation

An important consequence of GPVI downstream signaling, which serves to limit thrombus growth, is antibody or metalloproteinase (MMP) induced shedding of a 55 kDa GPVI ectodomain into the blood stream, leaving a 10 kDa remnant that remains platelet-associated.^22–24^ This plasma soluble form of GPVI (sGPVI) has been the marker for many quantitative studies on platelet activation through GPVI. The transmembrane metalloproteinases with protease activity, ADAM10 and ADAM17, have been identified as two sheddases that cleave the extracellular portion of GPVI independently of each other under various stimuli including the binding of GPVI ligands.^25^ However, when antibody-mediated GPVI downregulation occurs, the catalyst for GPVI shedding appears to be independent of these MMPs, as antibody induced GPVI shedding occurred in mice depleted of ADAM10 and ADAM17.^25^ Thus, it is likely that more sheddases participate in GPVI shedding and their role in stroke as a therapeutic target is yet to be determined.^26^

## GPVI and ischemic stroke

The importance of GPVI in hemostasis was first reported in a Japanese patient deficient in GPVI who had mild bleeding tendency and whose platelets failed to aggregate in response to collagen.^27^ Since then it has been found that platelets treated with GPVI-specific inhibitory antibodies show no interaction with collagen in vivo;^28^ In addition, GPVI-deficient individuals exhibit a mild bleeding tendency^28^ and GPVI-deficient mice show increased bleeding times.^30^ These observations cement GPVI's role as the main signal generator leading to platelet activation, rather than functioning primarily as a platelet adhesion receptor,^29^ but suggest that more work is needed to clarify the exact bleeding profile once GPVI is inhibited.

The exact role of GPVI in the different phenotypes of stroke is yet to be elucidated. We know that it plays a role in large artery atherosclerotic infarcts, but its role in lacunar and cardioembloic stroke is unclear. In large artery disease, elevated GPVI expression was shown to be associated with increased risk of stroke development. Enhanced GPVI expression is also seen after ischemic stroke and TIA, with these patients having a poorer clinical outcome at follow-up.^30^ Elevated sGPVI levels were found in patients specifically after large artery infarcts and these levels decrease after 3–6 months, which may highlight a role for sGPVI measurements in this stroke substrate, but the evidence in cardioembolic and lacunar types were less convincing.^31^

The pathogenesis of lacunar infarcts is believed to be due to progressive ischemic leukoaraiosis caused by a genetic susceptibility to inflammation-mediated cerebrovascular injury in combination with the classic atherosclerotic risk factors.^32^ There is evidence that chronic endothelial dysfunction and activation leading to a prothrombotic environment may cause progression of leukoaraiosis. This is evidenced by higher levels of prothormbotic proteins such as ICAM1, thrombomodulin, fibrinogen, tissue factor in patients with cerebral leukoaraiosis compared to controls.^33–35^ Recently, thrombogenic fibrin was shown to be an activator of GPVI in mice.^36^ Therefore, we could postulate that GPVI is involved in lacunar stroke and small vessel disease. Nevertheless, further studies are needed looking at GPVI in this subtype of stroke, where currently aspirin and clopidogrel play an important role in secondary prevention.

Recently, functional GPVI has been implicated as a possible receptor for polymerized fibrin, propagating thrombin generation.^37^ Further studies may reveal the role of GPVI in cardioembloic stroke as well as thrombus propagation, clot stabilization and infarct growth after ischemic stroke.^38^

Increased GPVI dimerization/multimerization could be one of the earliest measurable steps in platelet activation after plaque rupture. Studies are underway to measure GPVI dimer levels after acute stroke in comparison to healthy controls, which may be a useful diagnostic tool in the future. Since the dimer is the functional receptor form of GPVI, this may partially explain why the studies discussed above measuring total GPVI (dimers plus monomers) did not find any correlation between its levels and severity of stroke.^30^

## Targeting GPVI in ischemic stroke: Beyond antiplatelet therapy

Current antiplatelet therapy acts on this cascade of platelet activation via different methods and is licenced for both acute coronary syndrome (ACS) and ischemic stroke. Aspirin irreversibly inhibits both COX 1 and 2, thus stopping TxA2 formation after platelet activation. Clopidogrel and ticagrelor both irreversibly block the ADP receptor P2Y_12_.^39^ Thus both these classes of antiplatelet drugs work by inhibiting platelet aggregation. In comparison with single antiplatelet therapy, dual antiplatelets do convey a reduction in early stroke recurrence, combined transient ischemic attack (TIA), stroke and ACS, and all death^40^ but dual antiplatelet therapy is reserved for high-risk individuals after TIA or stroke due to the risk of hemorrhage.

Studies on whether other platelet glycoprotein receptors could be targets for antithrombotic therapy have yielded less promising results. Abciximab, the Fab fragment of a chimeric mouse/human monoclonal antibody, is licenced in ACS for patients awaiting percutaneous coronary intervention. It antagonizes GPIIb/IIIa, which is the final mediator in the pathway to platelet aggregation. Unfortunately it has shown no benefit in functional outcome in acute stroke, coupled with significant increases in fatal or symptomatic intracranial hemorrhage.^41^

Due to the fact that GPlbα (part of the GPIb-IX-V complex) plays a crucial role in platelet adhesion to endothelium in high shear conditions, it has become an attractive target for potential pharmaceutical development. GPlbα and GPVI are closely linked on the platelet surface and are thought to activate similar signaling processes.^42^ Much of the knowledge on platelet receptors and ischemic stroke has come from experimentation on animal models, particularly rats and mice, by causing transient middle cerebral artery occlusion (tMCAO). Blockade of GP1bα using Fab fragments, although reducing infarct volume after tMCAO, led to prolonged tail bleeding times compared to those treated with the monoclonal antibody (mAb) anti-GPVI JAQ1.^43^

From similar studies in mice, GPVI was shown to have a huge role in the formation of arterial thrombi. After tMCAO, GPVI-depleted mice (via JAQ1) demonstrate significantly reduced brain infarct volumes, no hemorrhagic transformation, normal platelet counts, and only moderately increased tail bleeding times.^28,43^ As platelets are anucleate cells and cannot synthesis protein de novo, injection of rat anti-GPVI antibodies (JAQ 1, 2, and 3) into mice in other studies offered long-term depletion of GPVI from murine platelet surfaces.^28,44^ Furthermore, the Fab fragments of antibodies 5C4,^45^ OM2,^46^ OM4,^47^ 9O12,^48^ and mFab-F^16^ are also potent inhibitors of GPVI-mediated platelet activation (Table 1). The evidence for antibody-driven GPVI blockade as a target for stroke therapy is increasing, with further efforts being made to attempt clearer translation into clinical medicine. Recently, Kraft et al.^49^ successfully showed that GPVI inhibition using JAQ 1 in adult mice with diabetes and hypertension resulted in smaller cerebral infarcts, better functional outcome, and reduced intracerebral hemorrhage rates compared to mice treated with anti-GPIIb/IIIa.^49^

**Table 1.** Antibodies that inhibit GPVI-mediated activation

	Type	Effects
JAQ 1^28^	Rat anti-mouse GPVI	Abolished platelet response to collagen or CRP Significantly reduced infarct size in tMCAO mice *Thrombocytopaenia and moderate bleeding-time prolongation*
9O12 (Fab)^48^	Mouse mAb against human GPVI	Protects against arterial thrombus propagation and collagen-induced aggregation in vitro and ex vivo *Mild bleeding-time prolongation*
5C4 (Fab)^45^	Rat mAb against human GPVI	Almost complete inhibition of platelet aggregation in vivo
OM2 (Fab)^46^	Mouse mAb against human GPVI	Inhibits collagen-induced platelet aggregation *Mild bleeding-time prolongation*
OM4 (Fab)^47^	Mouse mAb against human GPVI	Inhibits collagen-induced platelet aggregation in vitro and ex vivo Reduces in vivo thrombosis in rat carotid artery model *No bleeding-time prolongation*
10B12^58^	Human scFv against human GPVI	Inhibits CRP- and collagen-induced platelet aggregation in vitro
m-Fab-F^16^	Human Fab against human GPVI	GPVI dimer specific Inhibits aggregation and platelet adhesion on collagen

Patients with autoantibodies to GPVI-FcRγ chain, causing its clearance from the platelet surface are seen to have inefficiently formed thrombi on collagen-coated surfaces.^50^ However, their clinical presentation of mild thrombocytopenia, ecchymosis, epistaxis, and prolonged bleeding times highlights the difficulty in translating experimental findings into clinical practice.

Building on the work initially done on animal models,^45^ Revacept (PR-15, GPVI-Fc), a recombinant, soluble fusion protein between the human extracellular collagen-binding domain of GPVI and the C-terminal of human Fc, has been developed to competitively inhibit GPVI binding sites on exposed collagen. It was tested in a phase I trial, after intravenous administration to 30 healthy males, in 2011^51^ and showed clear dose-dependent inhibition of collagen responsiveness and platelet aggregation. This was done without any significant effect on hemostasis and no significant thrombocytopenia.

Revacept appears to avoid bleeding tendencies by blocking both the GPVI and VWF-mediated platelet activation processes specifically at the site of collagen exposure, thereby avoiding prolongation of bleeding times as described with antibodies to GPVI.^28,43^ However, this has only been confirmed on trials in mice after left MCA lesions.^52^ Revacept also seems to exert an anti-atherosclerotic effect via vessel endothelial remodelling and a role as a primary preventative medication in the absence of plaque rupture may emerge.^53^ It is currently undergoing phase II trials on patients with symptomatic carotid artery stenosis.^54^

Losartan, the angiotensin II receptor blocker has previously shown anti-atherosclerotic effects by blocking inflammogenic mediators and also anti-aggregatory effects independent of its effects on hypertension.^55^ EXP3179, its active metabolite, selectively inhibits GPVI function and therefore platelet adhesion and aggregation both in vitro and in vivo.^56^ This occurs when losartan binds to the IgG-like domain of GPVI^57^ and this interaction, coupled with the fact that losartan has no effect on bleeding profiles, may be the basis of a therapeutic target in the future.

## Conclusion

The platelet–collagen interaction, and subsequent platelet activation and thrombus formation, can lead to devastating neurological damage via ischemic stroke. There is a growing body of evidence pointing towards the importance of platelet glycoprotein receptors in stroke due to their requisite role in initiating downstream signaling mechanisms leading to platelet activation and pathological thrombus generation. Current understanding of thrombus formation suggests that GPVI is one such crucial receptor and inhibition of its collagen-induced signaling would be a specific pharmacological target for ischemic stroke. However, most of the research has been carried out in murine experimental models and may not necessarily translate to bleeding-free therapy in humans. The current clinical trials that are underway on Revacept are promising and could lead the way to a better understanding of GPVI antagonism in humans. Smaller studies are being carried out looking at novel small molecule inhibitors of GPVI and if a successful entity is discovered, the next decade should see the development of efficacious and highly specific therapies in ischemic stroke that a have large safety margin.
